# The imaging features of ectopic spleen: which modality is more consistent? A cases series report and literature reviews

**DOI:** 10.3389/fonc.2024.1310394

**Published:** 2024-03-11

**Authors:** Mingyue Xiao, Jiayi Liang, Jie Ren, Rongqin Zheng, Lili Wu

**Affiliations:** Department of Ultrasound, Third Affiliated Hospital, Sun Yat-sen University, Guangzhou, China

**Keywords:** ectopic spleen, wandering spleen, splenosis, contrast-enhanced ultrasonography, contrast-enhanced ultrasound

## Abstract

Ectopic spleen (ES) is a rare condition. It is difficult to diagnose with conventional imaging modalities. In this case series, we presented the imaging features of three misdiagnosed ES cases in our hospital and previously reported cases to compare the consistency of enhancement patterns among different imaging modalities with varied phases. Finally, 22 cases were reviewed. We determined that variable arterial phase enhancement and persistent enhancement throughout the portal and delayed phases are present in contrast-enhanced ultrasound (CEUS) imaging of the ES and found the arterial phase of CEUS had the highest consistency compared with computerized tomography (CT) and magnetic resonance imaging (MRI).

## 1 Introduction

Ectopic spleen (ES), which results from the autotransplantation of splenic tissue and often occurs after splenectomy, may be divided into two categories, namely, accessory spleens and wandering spleen, which are congenital foci of healthy splenic tissue that are distinct from the main body of the spleen (1, 2). Ectopic spleens usually present as asymptomatic conditions that may not require surgical resection. However, some ES may present as abdominal emergency and require medical intervention (3). The clinical symptoms of ES are usually nonspecific and related to the compression of another organ and torsion of the pedicle.

There is no definitive characterization by any imaging method (4). Therefore, the nonspecific clinical and imaging presentation makes the differential diagnosis of ES extremely challenging, and the final diagnosis is largely dependent on the pathological examination. In addition to the symptoms caused by ES itself requiring treatment, the overtreatment caused by misdiagnosis should not be ignored. Therefore, an accurate diagnosis of ES is crucial to its management.

It has recently been demonstrated that contrast-enhanced ultrasound (CEUS), which is employed in a variety of clinical situations, is useful for characterizing localized lesions (5, 6). Using the high accuracy of microbubble contrast agents, the blood flow around and inside the lesion was clearly visualized. The advantage of CEUS was especially useful in diagnosing liver lesions. In order to characterize ambiguous results of localized liver lesions after computed tomography (CT) and magnetic resonance imaging (MRI) scans, the European Federation of Societies for Ultrasound in Medicine and Biology (EFSUMB) has advocated using CEUS within its recommendations (5).

Therefore, we conducted this case series and literature reviews to summarize the ES imaging features of CT, MRI, and CEUS. Moreover, we tried to explore which imaging modality had the highest diagnostic consistency.

## 2 Case presentations

**Figure 1:**
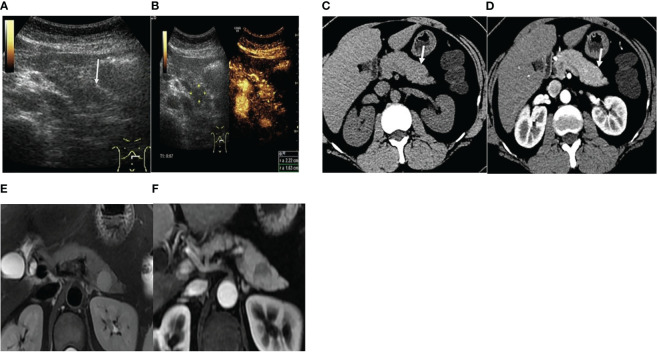
Case 1 in our hospital. **(A)** Conventional two-dimensional ultrasonography revealed an ill-defined suborbicular lesion in the pancreatic tail (white row), with uneven hypoecho, of approximately 2.2 cm in size. **(B)** CEUS revealed hyperenhancement in the arterial phase. **(C, D)** Contrast-enhanced CT demonstrated a round iso-dense lesion (white rows) in the tail of the pancreas, with the mild enhancement in the arterial phase. **(E)** T2WI sequence revealed a well-defined round mass in the pancreatic tail, with high intensity. **(F)** Contrast-enhanced MRI showed slightly delayed enhancement (white row).

Case 1 was a 34-year-old woman without a history of splenectomy or trauma. She had no clinical manifestations other than fatigue and weight gain. Conventional ultrasound (US) revealed a hypoechoic circular lesion in the tail of the pancreas, approximately 2.2 cm in size, with uniform internal echogenicity and clear borders, which means that no obvious Echo alteration can be observed and to distinguish the lesion with surrounding tissue is difficult (**Figure 1A**). CEUS suggested hyperenhancement in the arterial phase (**Figure 1B**) and mild hyperenhancement in the portal and delayed phases. There were three uniform solid masses with punctate blood flow signals seen in the liver, and CEUS showed equal or high enhancement in the arterial and portal phases and equal or low enhancement in the delayed phase. Enhanced CT suggested a round, isointense lesion in the tail of the pancreas (**Figures 1C, D**). MRI suggested a well-defined mass with low signal in the T1WI sequence and high signal in the T2WI sequence and a slight enhancement in the enhanced scan with late phase (**Figures 1E, F**). The patient also underwent positron emission computed tomography (PET) examination, and no obvious metabolic abnormality was found in the nodule of the pancreatic tail (**Supplementary Figure S1**). Based on the above data, the patient was diagnosed with pancreatic neuroendocrine tumor (PNET) with liver metastasis. Eventually, the patient subsequently underwent resection of the pancreatic and hepatic masses, splenectomy, and hepatic mass microwave ablation. Pathological results identified the lesion was ES.

**Figure 2:**
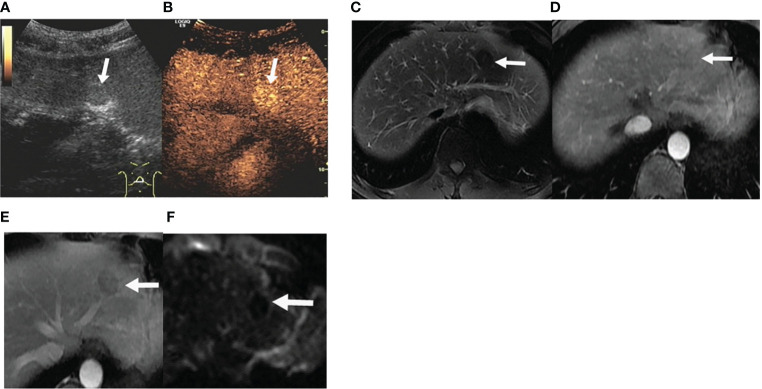
Case 2 in our hospital. **(A)** US demonstrated a round mass in the segment II of the liver near the dome of the diaphragm, with even slightly hyperecho, measuring about 1.9 cm ×1.6 cm. **(B)** CEUS was performed and revealed hyperenhancement in the delayed phase (white rows). **(C)** The mass in the segment II of the liver (white rows) revealed low T2 signal. **(D, E)** Enhanced MR showed an arterial enhancement and had a gradual washout in the venous phases. **(F)** On DWI, no restricted diffusion was shown in the mass.

Case 2 was a 39-year-old man who had a splenectomy 16 years ago. He experienced no clinical symptoms. US suggested a slightly hyperechoic round mass in segment II of the liver measuring approximately 1.9 cm in size with even internal echogenicity and clear borders (**Figure 2A**). CEUS suggested high enhancement in the arterial, portal, and delayed phases (**Figure 2B**). MRI examination suggested that the mass showed low T1 signal intensity and low T2 signals (**Figures 2C–E**). Magnetic resonance diffusion-weighted imaging (DWI) showed no restricted diffusion in the mass (**Figure 2F**). Based on the above imaging findings, it is suspected of atypical vasoproliferative tumor of the liver or focal nodular hyperplasia (FNH). Subsequently, laparoscopic resection of the tumor located in liver segment II was performed. Pathological results identified that the lesion was ES.

**Figure 3:**
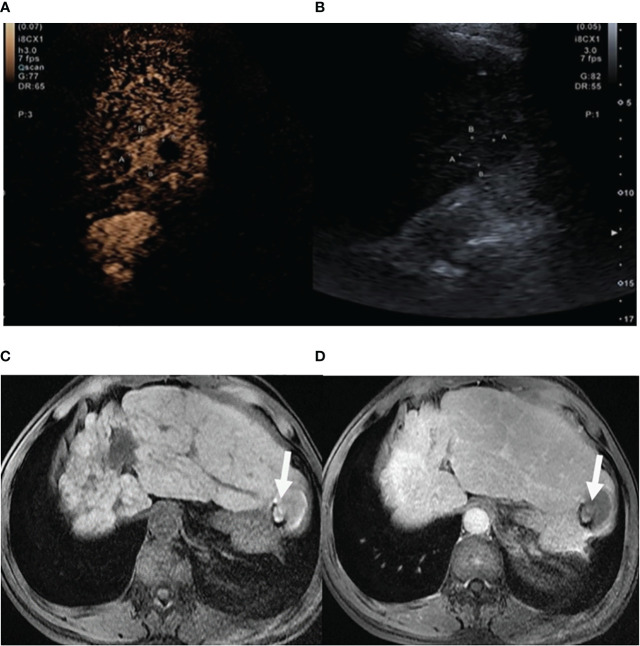
Case 3 in our hospital. **(A)** CEUS suggested iso-enhancement in the portal phase (white rows). **(B)** Routine two-dimensional US showed an iso-echoic and round-like mass in the segment II of the liver. **(C, D)** The lesion (white rows) showed short T2 signal, and the enhancement scan suggested gradual signal attenuation after 5 min in the delayed phase.

Case 3 was a 57-year-old man with a history of chronic hepatitis B cirrhosis. He had three hepatocellular carcinoma ablations and splenectomy 4 years prior with no clinical symptoms. Two-dimensional US showed an isoechoic and round-like mass in segment II of the liver, approximately 1.9 cm in size. The boundary was unclear, and the internal echo was even. CEUS suggested hyperenhancement in the arterial phase and iso-enhancement in the portal and delayed phases (**Figures 3A, B**). MRI and the enhancement scan are shown in **Figures 3C, D**.

The baseline and imaging data for the three cases are shown in **Table 1**, and the pathological presentations are detailed in **Supplementary Figures S2**-**S4**. Pathological results identified that the lesion was ES.

**Table 1:** The basic characteristics of three patients with ectopic spleens.

	Case 1	Case 2	Case 3
Sex/age(years)	F/34	M/39	M/57
History of surgery	No	Splenectomy	Splenectomy
Clinical presentation	Asymptomatic	Asymptomatic	Asymptomatic
Contrast-enhanced CT	High density	–	–
MRI			
T1W1	Low signal intensity	Low signal intensity	Low signal intensity
T2W2	High signal intensity	Low signal intensity	High signal intensity
Contrast-enhanced MRI			
Arterial phase	–	Enhancement	Enhancement
Portal phase	–	Enhancement	Enhancement
Venous phase	–	Attenuation	Enhancement
Delayed phase	Slightly enhancement	Attenuation	Attenuation after 5 min
CEUS			
Arterial phase	High	High	High
Portal/venous phase	Slightly high	High	Isoechoic
Delayed phase	Slightly high	High	Isoechoic
Preoperative Diagnosis	PNET with liver metastases	FNH	Abnormal perfusion/HCC
Postoperative Diagnosis	Ectopic spleen	Ectopic spleen	Ectopic spleen

F, female; M, male; CEUS, contrast-enhanced ultrasound; PNET, pancreatic neuroendocrine tumor; FNH, focal nodular hyperplasia; HCC, hepatocellular carcinoma.

## 3 Case summaries from previous studies

After the databases were searched, the data for 22 cases who underwent CT, MRI, and CEUS from 17 case reports were obtained (7–23). In total, 25 cases were included for further analysis. The summary of the baseline characteristics of the 25 cases is presented in **Table 2**. Of the patients, 83.3% of them were men, with a median age of 53 years (ranging from 10 to 70 years); 75% (12/16) had a history of splenectomy, and one case had splenic trauma. The sizes of the lesions ranged from 0.5 cm to 7 cm and were unknown in one case; the median tumor size was 2.1 cm. Most (22/25, 88%) of the lesions were single and often located in the pancreatic tail (48%, 12/25), the liver (20%, 5/25), the intraperitoneum, or the abdominal cavity organs (16%, 4/25), and so on.

**Table 2:** Baselines characteristics of the 25 cases with ectopic spleen.

Source, year	Case	Sex	Age	History of splenectomy	Size, cm	Location
Ota et al. (7), 2004	1	M	31	NI	NI	Pancreatic tail
Kim et al. (8), 2005	2	M	45	NI	1.7	Pancreatic tail
	3	M	60	NI	1.8	Pancreatic tail
	4	M	70	NI	1.3	Pancreatic tail
	5	M	32	NI	2.4	Pancreatic tail
	6	F	70	NI	1.1	Pancreatic tail
	7	F	43	NI	1.2	Pancreatic tail
Ota et al. (9), 2005	8	M	39	Yes	2.0	Intra peritoneum
Ferraioli et al. (10), 2006	9	M	40	Yes	6.0	Liver
Rogers et al. (11), 2011	10	M	64	Yes	5.0	Pancreatic Tail
Makino et al. (12), 2011	11	M	59	NI	1.3	Pancreatic Tail
De Robertis et al. (13), 2014	12	M	53	Yes	2.8	Pancreatic Head
Marques et al. (14), 2016	13	M	69	NI	0.5	Pancreatic Tail
Kruger and Freeman (15), 2018	14	M	56	Yes	4.3	Pelvic Mass
Sansone et al. (16), 2020	15	M	46	Yes	4.9^#^	Liver
Luo et al. (17), 2020	16	F	49	No	2.7	Pancreatic Tail
Dölle et al. (18), 2021	17	M	62	No^*^	2.6	Liver
Zhong et al. (19), 2021	18	M	55	Yes	5.3^#^	Liver
Yankov (20), 2022	19	M	10	No	1.3	Pancreatic body
Foh et al. (21), 2022	20	M	63	Yes	5.6^#^	Thoracic masses/abdominal masses
Kroenig et al. (22), 2022	21	NI	54	Yes	7.0	Pleural mass
Liu et al. (23), 2022	22	M	39	Yes	2.0	Liver
Our case 1	23	F	34	No	2.2	Pancreatic tail
Our case 2	24	M	39	Yes	1.9	Abdominal mass and liver
Our case 3	25	M	57	Yes	1.9	Abdominal mass and liver

*Have a history of splenic trauma.

^#^There were at least three lesions in the case, and the size of the largest lesion is listed in the table.

NI, no information; M, male; F, female.

### 3.1 The presentations of CT and MRI

The most common presentations of enhanced CT in the arterial phase were homogeneous high enhancement (10/19; 52.6%), followed by inhomogeneous high enhancement (6/19; 31.6%). On portal and late phases, the most common findings of masses were homogeneous high enhancement (66.7%, 12/18) (**Table 3**). On the arterial phase, the most common presentations of MRI scans were homogeneous high enhancement (8/15; 53.3%), followed by inhomogeneous high enhancement (4/15; 26.7%) (**Table 3**).

**Table 3:** CT and MRI presentations of the 25 cases with ectopic spleen.

Case	CT presentations			MRI presentations		
	Arterial Phase	Portal Phase	Late Phase	Arterial Phase	Portal Phase	Late Phase
1	NI	NI	NI	NI	NI	NI
2	IE	HE	HE	IE	HE	HE
3	HE	HE	HE	HE	HE	HE
4	IE	HE	HE	IE	HE	HE
5	HE	HE	HE	HE	HE	HE
6	IE	HE	HE	IE	HE	HE
7	HE	HE	HE	HE	HE	HE
8	HE	HE	HE	NI	NI	NI
9	NI	NI	NI	NI	NI	NI
10	IE	HE	HE	NI	NI	NI
11	HE	IE	HI	NI	NI	NI
12	HE	HE	HE	HE	HE	HE
13	NI	NI	NI	NI	NI	NI
14	HE	HE	HE	HE	HE	HE
15	NI	NI	NI	HE	HE	HE
16	LE	LE	LE	NI	NI	NI
17	NI	NI	NI	HE	HE	HE
18	IE	IE	IE	NI	NI	NI
19	NI	NI	NI	HI	HI	HI
20	HE	NI	NI	NI	NI	NI
21	HE	HE	HE	NI	NI	NI
22	IE	LE	LE	IE	HI	HI
23	HE	HE	HE	HE	HE	HE
24	HI	HI	HI	HI	HI	HI
25	HI	HI	HI	HI	HI	HI

IE, inhomogeneous high enhancement; HE, homogeneous high enhancement; HI, homogeneous isoenhancement; NI, no information, LE, low enhancement.

### 3.2 The presentations of CEUS

Compared with the surrounding normal tissue, 91% (20/22) of the lesions showed hyperenhancement in the arterial phase. Most of the lesions showed varying degrees of hyperenhancement in the portal phase and delayed phase, respectively. In the delayed phase, only three cases showed iso-enhancement or obvious washout (hypoenhancement). A total of 11 cases described the degree of enhancement of the lesion compared with the splenic parenchyma, all of which presented equal enhancement at different phases, showing a similar enhancement pattern to that of the spleen (**Table 4**).

**Table 4:** Contrast-enhanced ultrasonography presentations of the 25 cases with ectopic spleen.

Case	Echo Enhancement Compared with Surrounding Tissues			Echo Enhancement Compared with Spleen
	Arterial Phase	Portal/Venous Phase	Delayed Phase	
1	NI	NI	Low	Isoechoic (delayed phase)
2	High	High	High	Isoechoic (three phases)
3	High	High	High	Isoechoic (three phases)
4	High	High	High	Isoechoic (three phases)
5	High	High	High	Isoechoic (three phases)
6	High	High	High	Isoechoic (three phases)
7	High	High	High	Isoechoic (three phases)
8	NI	NI	High/Moderate	NI
9	Low	High	High	Isoechoic (three phases)
10	High	High	High	NI
11	High	High	NI	Isoechoic (arterial phase and venous phase)
12	High	High	High	Isoechoic (delayed phase)
13	High	NI	NI	NI
14	High	High	High	Isoechoic (delayed phase)
15	High	High	High	NI
16	Ring-shaped High	Ring-shaped High	Ring-shaped High	NI
17	High	High	High	NI
18	High	High	High	NI
19	Low	Isoechoic	Isoechoic	NI
20	High	High	High	NI
21	NI	High	High	NI
22	High	Slightly high	Slightly high	NI
23	High	Slightly high	Slightly high	NI
24	High	High	High	NI
25	High	Isoechoic	Isoechoic	NI
	High, 20/22 (91%)	High, 20/22 (91%)	High, 20/23 (87%)	Isoechoic, 11/11(100)

NI indicates no information.

### 3.3 Comparison results of different imaging modalities

On the arterial phase, the enhancement patterns of lesions in CEUS (91%) presentations had higher consistency than CT (52.6%) and MRI (53.3%), and the differences were significantly different (p = 0.017 and 0.012). On portal phase, the enhancement patterns in CEUS (91%) presentations had higher consistency than CT (66.6%) and MRI (73.3%), but the differences were not significantly different (p = 0.10 and 0.198). Similarly, on the delayed phase, the enhancement patterns in CEUS (87%) presentations had higher consistency than CT (66.6%) and MRI (73.3%), but the differences were not significantly different (p = 0.147 and 0.401, respectively).

## 4 Discussion

ES is rare in clinical practice. It usually relates to spleen trauma or splenectomy, which could cause the direct spread of splenic tissue fragments. It might also occur along the splenic vessels, in the splenorenal or gastrosplenic ligaments, the greater omentum or the mesentery, the wall of the stomach or bowel, and in the pelvis or scrotum (24). ES usually involves multiple lesions, ranging in diameter from a few millimeters to 7 cm and even >12 cm (25). It is impossible to perform surgical exploration in every single patient; therefore, imaging examinations are still the first choice in diagnosing ES. The ability to diagnose ES is crucial for avoiding unnecessary surgery. The variable morphology and clinical manifestations made it difficult to diagnose.

As a result of the varying flowrates of the cord and sinuses in the red pulp, CT or MRI of a normal spleen may show a mottled pattern of enhancement in the arterial phase and early portal venous phase (26, 27). However, the implanted splenic tissue lacks the usual splenic vascular pattern, and the blood supply pattern is different. In addition to our three cases, other rare case reports demonstrate that ectopic spleen is usually misdiagnosed as a malignant or benign hypervascular neoplasm with nonspecific imaging features on US, CT, or MRI (14–16). In our study, CT and MRI had a diagnostic consistency of 50%–70% in diagnosing ES; therefore, sometimes CT and MRI are not reliable.

CEUS, a safe, nonradiative, relatively inexpensive, and convenient imaging modality, can provide useful evidence for clinical diagnosis. CEUS applications have gained increasing experience in the differential diagnosis of space-occupying lesions in recent years. Numerous national and international groups have advocated the use of the CEUS test for the diagnosis of focal liver lesions (28). Referring to the CEUS performance of ES, it is rarely reported in the literature.

According to reports, SonoVue is a pure blood pool contrast agent, meaning that after injection, the microbubbles only temporarily stay in the blood vessel. A pharmacological kinetics study showed that the intake of the contrast agent in the right hepatic lobe and both kidneys declined from 88% to 67% within 5 min but that the spleen practically did not experience the same phenomenon (90%–99% intake) (29). Despite the fact that the mechanism behind how long the contrast microbubbles are kept in the spleen parenchyma or if they are also phagocytized by macrophages is yet unknown, SonoVue results in a spleen-specific enhancement that lasts for up to 5 min. This is distinctive and has a particular significance for the ES diagnosis.

Both benign and malignant neoplasms might exhibit hyperenhancement in the arterial phase. However, portal venous blood volume of malignant tumors is lower, which causes the contrast agent to wash out in the portal and/or late phases. As a result, for benign lesions, enhancement often continues until the late phase (29). Our study also identified that hyperenhancement in the portal or delayed phase was more common than arterial enhancement in CT and MRI presentations. Due to the small sample size, the statistical differences were not confirmed. However, we could still conclude that the hyperenhancement in the venous and delayed phase, a typical feature of most benign lesions, provides a clue for diagnosing ES, especially in patients who have undergone splenectomy or splenic trauma.

The three cases at our center showed the R2* value ranging from 225 to 275 in the IDEAL-IQ sequence, which is a quantitative indicator of iron deposition, indicating iron overload in our cases. Histology may provide evidence to support the theory that this is a result of splenic reticuloendothelial cells phagocytosing iron particles (30). Thus, lesions on T2WI sequences that exhibit equal or low signal intensity may aid in the confirmation of the diagnosis of ES. However, Berlin blue staining was not performed on all of the case reports included, and the feature should be validated in future studies.

In our study, we determined that variable arterial phase enhancement and persistent enhancement throughout the portal and delayed phases are present in CEUS imaging of the ES. We also identified that the consistency of enhancement patterns was higher in CEUS than CT or MRI, especially in the arterial phase. Our study may greatly benefit from the knowledge of CEUS in the management of ES.

## Data availability statement

The raw data supporting the conclusions of this article will be made available by the authors, without undue reservation.

## Ethics statement

The studies involving humans were approved by Ethics Committee of the Third Affiliated Hospital of Sun Yat-sen University. The studies were conducted in accordance with the local legislation and institutional requirements. Written informed consent for participation was not required from the participants or the participants’ legal guardians/next of kin in accordance with the national legislation and institutional requirements. Written informed consent was obtained from the individual(s) for the publication of any potentially identifiable images or data included in this article.

## Author contributions

MX: Data curation, Writing – original draft. JL: Formal Analysis, Methodology, Writing – original draft. JR: Supervision, Writing – review & editing. RZ: Supervision, Writing – review & editing. LW: Supervision, Writing – review & editing.
