# Supplementary material for: The imaging features of ectopic spleen: which modality is more consistent? A cases series report and literature reviews
Source: Front Oncol. 2024 Mar 11;14:1310394. doi: 10.3389/fonc.2024.1310394 (PMC10961413; doi:10.3389/fonc.2024.1310394)
Supplement: Supplementary file 1 [file Image_1.pdf]

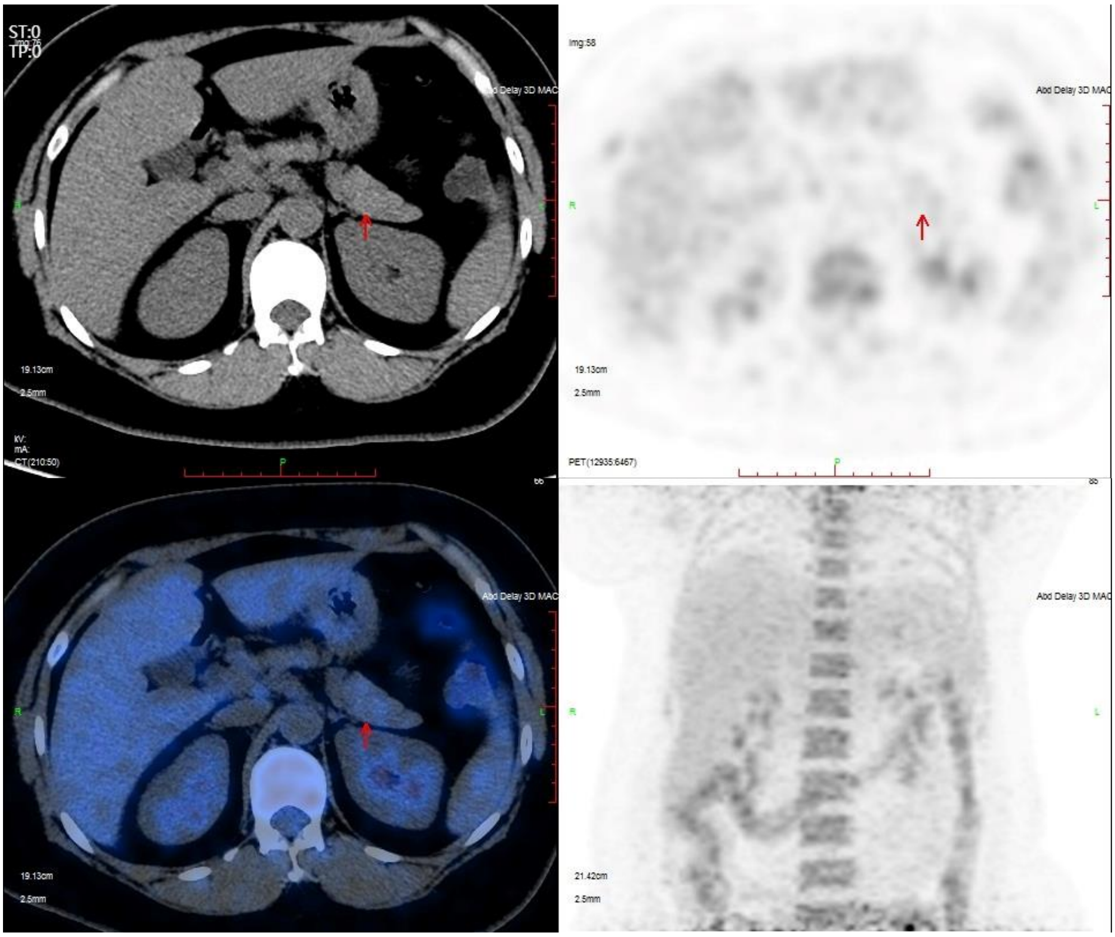

Supplementary Figure 1. PET examination of Case1 in our hospital. No obvious metabolic abnormality was found in the nodule of the pancreatic tail, and the nature was undetermined. There were no obvious abnormal metabolic lesions in the liver.

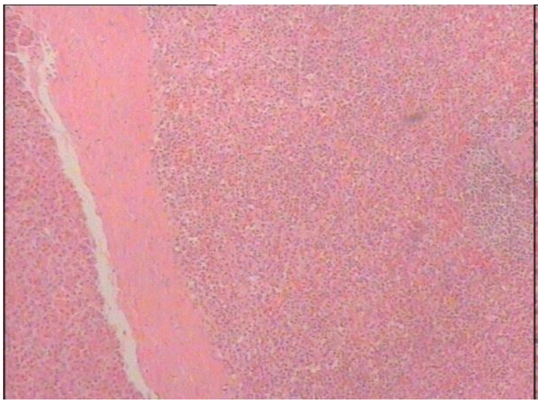

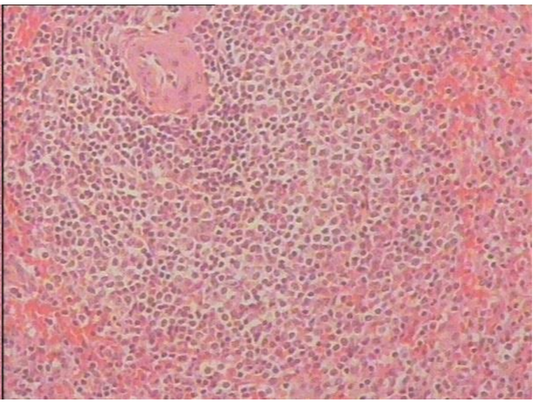

2A 2B

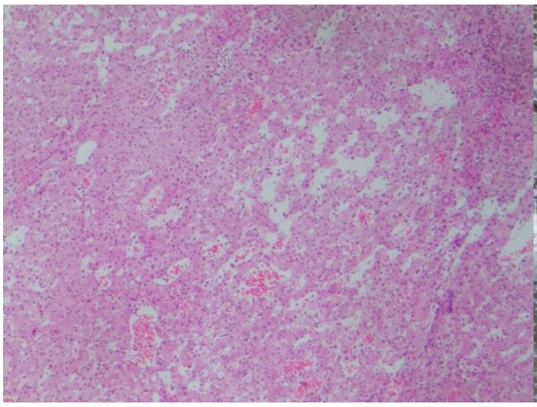

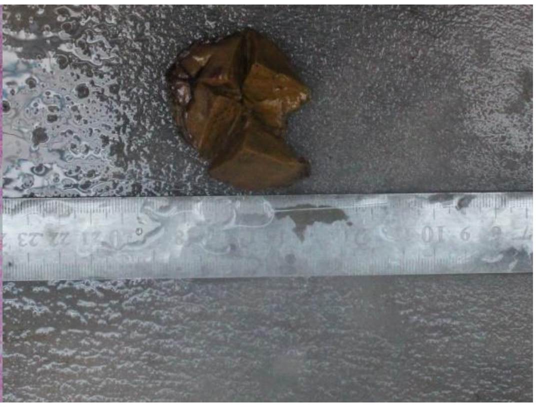

2C 2D

Supplementary Figure 2. Case1 in our hospital. There is a 4*4*2cm gray pancreas tail, 1.5*1.5cm dark red mass can be seen when incised, with clear boundary and dark red matter in the section surface. Ectopic splenic tissue was found in the pancreas. Most of the liver lobular mass in the left extrahepatic lobe was normal with hepatocyte steatosis and adenomatous hepatocyte hyperplasia in some areas.

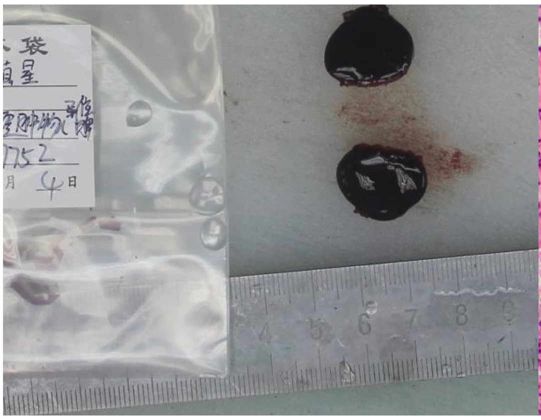

3A

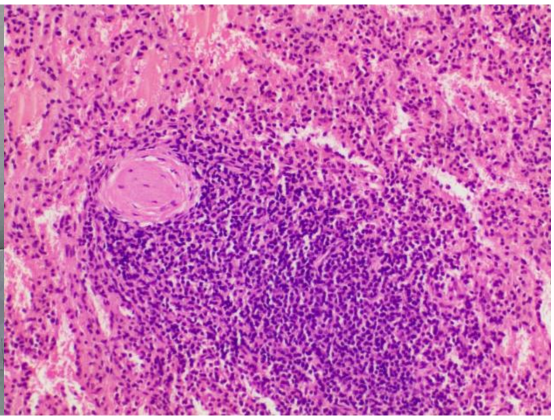

3B

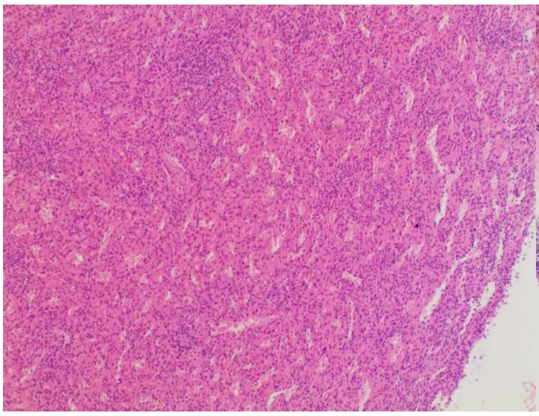

3C

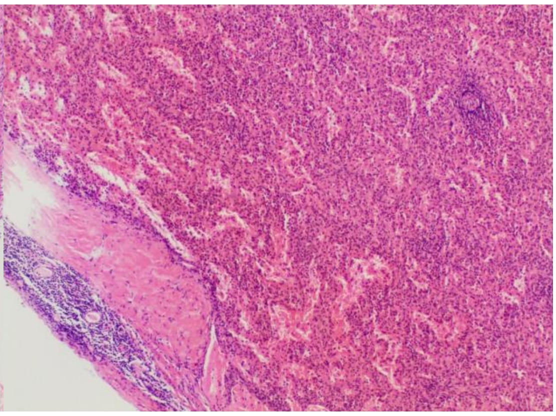

3D

Supplementary Figure 3. Our case 2. A 2*1.5*1.5cm grayish-red tissue was observed, and the structure of red pulp and white pulp was seen under the microscope.

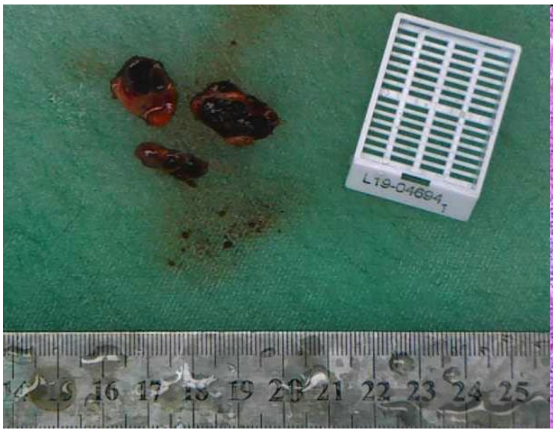

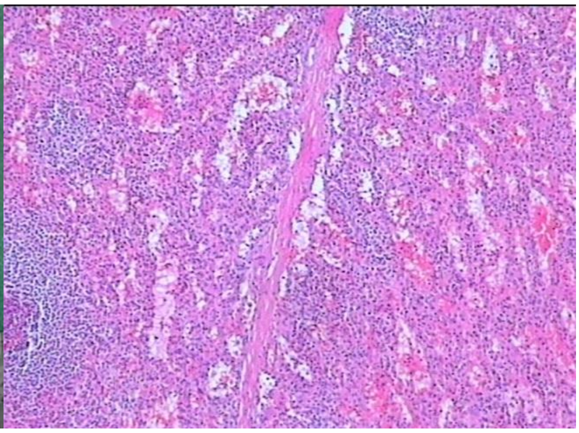

4A 4B

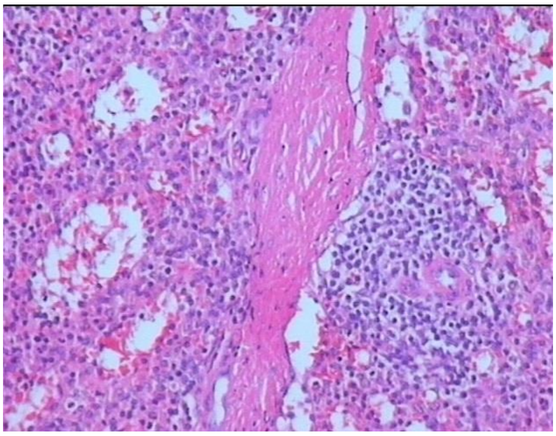

4C

Supplementary Figure 4. Our case 3. One gray-red nodule (1.8*1.3*0.8cm) was observed. Microscopically, the mass was nodular, with many lacune-like structures accompanied by congestion and hemorrhage, and a small amount of scattered lymphoid tissue gathered into foci around small arteries.
